# Refined fiber inulin promotes inflammation‐associated colon tumorigenesis by modulating microbial succinate production

**DOI:** 10.1002/cnr2.1863

**Published:** 2023-07-25

**Authors:** Sangshan Tian, Devendra Paudel, Fuhua Hao, Rabin Neupane, Rita Castro, Andrew D. Patterson, Amit K. Tiwari, K. Sandeep Prabhu, Vishal Singh

**Affiliations:** ^1^ Department of Nutritional Sciences The Pennsylvania State University University Park Pennsylvania USA; ^2^ Department of Veterinary and Biomedical Sciences The Pennsylvania State University University Park Pennsylvania USA; ^3^ Department of Pharmacology and Experimental Therapeutics University of Toledo Toledo Ohio USA

**Keywords:** colon cancer, gut microbiota, inflammatory bowel disease, microbial metabolism, nutrition, prebiotic fiber

## Abstract

**Background and Aim:**

There is an increased risk of colon cancer associated with inflammatory bowel disease (IBD). Dietary fibers (DFs) naturally present in vegetables and whole grains offer numerous beneficial effects on intestinal health. However, the effects of refined DFs on intestinal health remain unclear. Therefore, we elucidated the impact of the refined DF inulin on colonic inflammation and tumorigenesis.

**Methods:**

Four‐week‐old wild‐type (WT) mice were fed diets containing insoluble DF cellulose (control) or refined DF inulin for 4 weeks. A subgroup of mice was then switched to drinking water containing dextran sulfate sodium (DSS, 1.4% wt/vol) for colitis induction. In another subgroup of mice, colitis‐associated colorectal cancer (CRC) was initiated with three 7‐day alternate cycles of DSS following an initial dose of mutagenic substance azoxymethane (AOM; 7.5 mg/kg body weight; i.p.). Post 7 weeks of AOM treatment, mice were euthanized and examined for CRC development.

**Results:**

Mice consuming inulin‐containing diet exhibited severe colitis upon DSS administration, as evidenced by more body weight loss, rectal bleeding, and increased colonic inflammation than the DSS‐treated control group. Correspondingly, histological analysis revealed extensive disruption of colon architecture and massive infiltration of immune cells in the inulin‐fed group. We next examined the effect of inulin on CRC development. Surprisingly, significant mortality (~50%) was observed in the inulin‐fed but not in the control group during the DSS cycle. Consequently, the remaining inulin‐fed mice, which completed the study exhibited extensive colon tumorigenesis. Immunohistochemical characterization showed comparatively high expression of the cell proliferation marker Ki67 and activation of the Wnt signaling in tumor sections obtained from the inulin‐fed group. Gut microbiota and metabolite analysis revealed expansion of succinate producers and elevated cecal succinate in inulin‐fed mice. Human colorectal carcinoma cells (HCT116) proliferated more rapidly when supplemented with succinate in an inflamed environment, suggesting that elevated luminal succinate may contribute to tumorigenesis.

**Conclusions:**

Our study uncovers that supplementation of diet with refined inulin induces abnormal succinate accumulation in the intestinal lumen, which in part contributes to promoting colon inflammation and tumorigenesis.

## 1 INTRODUCTION

Colorectal cancer (CRC) is the third most common cancer among all cancer types in the United States and the fourth highest worldwide.^1^ Both genetic (non‐modifiable) and environmental (modifiable) risk factors play a role in CRC development. Recurring episodes of mucosal inflammation in patients with inflammatory bowel disease (IBD) increase the risk of CRC development in the long run.^2^, ^3^ Therefore, controlling and reducing intestinal inflammation in IBD patients is viewed as a potential strategy to prevent the initiation and development of gastrointestinal (GI) malignancies, including CRC. IBD is a multifactorial pathology that results from genetic mutations alone or in combination with detrimental changes in environmental factors, including the composition and activity of the intestinal microbiota, which collectively heightens intestinal immune activity.^4^, ^5^ The two clinical forms of human IBD, Crohn's disease (CD) and ulcerative colitis (UC), result from chronic GI inflammation and are initiated by dysregulated intestinal immune responses.

Dietary fibers (DFs) are plant‐derived complex carbohydrates that influence the richness of the bacterial species and their metabolites in the gut.^6^ In fact, the degree of solubility and chemical structures of DFs and the ability of bacteria to degrade these DFs determine whether gut bacteria will utilize a specific DF type to fulfill their energy and nutrient needs.^7^ For example, cellulose is an insoluble polysaccharide of D‐glucose [β‐(1,4)‐linked D‐glucose] units^8^ that resists intestinal bacterial fermentation in humans and mice.^9^ Thus, cellulose is considered to be a non‐fermentable fiber, particularly in humans and mice. In contrast, inulin, made of β‐(2,1)‐linked fructose, is easily degraded by the human and mouse gut microbiota, mainly by *Bifidobacteria* and *Bacteroides*.^10^ Thus, inulin is classified as a fermentable dietary fiber (FDF), and is widely used in the food industry to improve the texture of reduced‐fat products and as a sugar substitute in processed foods.^11^

Many studies have demonstrated that gut microbiota dysbiosis, largely defined by reduced microbial richness or expansion of certain species, negatively influences overall health. Reduced microbial diversity due to low consumption of FDFs has been linked to poor intestinal and metabolic health.^12^, ^13^ Therefore, incorporation of FDFs in processed foods is being advocated to improve overall health by correcting such dysbiosis and promoting the growth of beneficial bacteria. In support, clinical and pre‐clinical studies suggest that inulin consumption by healthy individuals improves the gut barrier function and production of beneficial fermentation metabolites such as short‐chain fatty acids (SCFAs).^14^, ^15^, ^16^, ^17^ In this effort, food manufacturers incorporate refined inulin as prebiotics into food products to promote the growth of specific groups of bacteria, which are believed to be beneficial for human health. However, recent research cautions that FDFs might not be universally beneficial for health^18^, ^19^, ^20^ and whether such refined fiber‐enriched food products deliver the expected health benefits during dysbiosis remain to be ascertained. We hypothesize that the effects of FDFs on GI inflammation and malignancies depend on the intestinal inflammatory state, the capability of gut microbiota utilizing the consumed FDF, and metabolites resulting from microbial fermentation. An elegant study conducted by Armstrong HK et al.^20^ revealed that the presence of unfermented inulin increases the inflammatory response in a subset of IBD patients with reduced fermentative capacity, supporting the premise that the effect of FDF on intestinal health is context‐dependent and linked with the fermentative capability of residing microbes in the intestine.

In the present study, we investigated the effect of refined inulin, a FDF believed to have overall health‐promoting effects, on colonic inflammation and carcinogenesis. Specifically, we fed mice with inulin‐containing diet or control diet containing the non‐fermentable fiber cellulose. The effects of inulin on intestinal inflammation were assessed by using the dextran sulfate sodium (DSS)‐induced colitis model and on colon tumorigenesis by employing the azoxymethane (AOM)/DSS model of CRC. DSS is a widely used classic colitogenic substance that causes damage to the mucosal barrier and epithelial cells to trigger intestinal immune response, preferably in the colon, by exposing the commensal microorganisms to lamina propria cells.^21^, ^22^

## 2 METHODS

### 2.1 Animal model and experimental diets

Inbred four‐week‐old wild‐type (WT, C57BL/6J) mice were housed in a temperature‐ and humidity‐controlled room with free access to food (Laboratory Rodent Diet 5001) and water. One‐week post‐weaning experimental mice were switched to isocaloric diets containing cellulose (10% wt/wt, control diet, Con) or inulin (7.5% wt/wt inulin plus 2.5% wt/wt cellulose; Inu) (Research Diets, Inc., New Brunswick, NJ). The composition of the experimental diets is shown in Table 1. Diets were replaced once a week. All procedures were approved and performed in compliance with the Institutional Animal Care and Use Committee (IACUC) of The Pennsylvania State University. Both male and female mice were used in this study. Since there was no difference in colitis susceptibility, we combined the data from both sexes.

**TABLE 1:** Diet composition.

Diet formulas	Cellulose‐containing diet (Con)		Inulin containing diet (Inu)	
**Product #**	**D12081402**		**D12081401**	
	gm%	kcal%	gm%	kcal%
Protein	18.5	20	19	20
Carbohydrate	61.8	65	67.8	65
Fat	6.4	15	6.5	15
Total		100		100
kcal/gm		3.78		3.88
**Ingredients**	**gm**	**kcal**	**gm**	**kcal**
Casein, 30 mesh	200	800	200	800
L‐cysteine	3	12	3	12
Corn starch	409	1636	381	1524
Maltodextrin 10	110	440	110	440
Dextrose	150	600	150	600
Cellulose, BW200	100	0	25	0
Inulin (Orafti® HP)	0	0	75	113
Soybean oil	70	630	70	630
Mineral mix S10026	10	0	10	0
Dicalcium phosphate	13	0	13	0
Calcium carbonate	5.5	0	5.5	0
Potassium citrate	16.5	0	16.5	0
Vitamin mix V10001	10	40	10	40
Choline bitartrate	2	0	2	0
FD&C red dye #40	0	0	0.05	0
FD&C blue dye #1	0.05	0	0	0

#### 2.1.1 Dextran sulfate sodium‐induced acute colitis

WT mice were weaned at day 22 (±1 day) and maintained on the standard chow diet. After 1 week, mice were switched to one of the experimental diets (*n* = 4 in each group). After 4 weeks, DSS (1.4%, wt/vol) was added to the drinking water. Body weight was monitored daily. Seven days post‐DSS intervention, the animals were euthanized by carbon dioxide, and blood, colon, and cecal samples were collected as described below and stored at −80°C until further analysis. Con or Inu‐fed mice maintained on regular water were used as basal‐level control groups.

#### 2.1.2 Colitis‐associated cancer induction

Four‐week‐old WT mice (*n* = 8–12) were maintained on experimental diets for 3 weeks before cancer induction. Subsequently, colon tumorigenesis was induced by azoxymethane (AOM, Sigma‐Aldrich)‐DSS administration, as previously described in detail.^23^ Briefly, AOM was injected intraperitoneally (7.5 mg/kg body weight). After 7 days, colonic inflammation was induced by the cycle of seven‐day DSS water and 7‐day regular water. The DSS concentrations in the drinking water were 1% wt/vol in the first cycle and 0.75% wt/vol in the second and third cycles. The mice were euthanized 14 days post‐last DSS cycle. The body weight of the animals was collected every 3 days.

### 2.2 Sample collection and preparation

#### 2.2.1 Serum

Blood was collected from the euthanized mice into serum‐separation tubes (BD Microtainer). Serum was then obtained by centrifugation at 700*g* for 8 min at 4°C and stored at −80°C until further analysis.

#### 2.2.2 Colon

Colons were dissected and flushed with ice‐cold PBS. The proximal part (~1.5 cm) was stored at −80°C until protein extraction. With the remaining colon tissue, a Swiss roll formed from the proximal to the distal end and was fixed in 10% neutral buffered formalin (NBF, Fisher Chemical) for 24 h, then stored in 70% ethanol until embedded in paraffin for histology analysis.

#### 2.2.3 Protein extraction and quantification

Colon proteins were extracted with RIPA Lysis and Extraction Buffer (Thermo Scientific) by adding 1% protease inhibitor (Thermo Scientific) and 1% phosphatase inhibitor (Thermo Scientific). The total protein content was quantified according to the manufacturer's instructions using Pierce™ bicinchoninic acid protein assay kit (Thermo Scientific).

### 2.3 Inflammatory markers quantification

Serum and colon lipocalin 2 (Lcn2) and serum amyloid A (SAA) levels were measured by ELISA (R&D systems) as per the manufacturer's instructions.

### 2.4 Histochemical analysis

Colonic sections (5 μm thickness) were obtained from paraffin‐embedded colon tissue (Animal Diagnostic Laboratory, The Pennsylvania State University). To perform the staining, the colon sections were deparaffinized by immersing in xylene, gradient ethanol (100%, 90%, 70%, and 50% vol/vol), and PBS with the help of Leica ST5010 autostainer (Leica Microsystems Inc.). Hematoxylin and eosin (H&E)‐stained sections were used to examine the changes in crypt structure, ulceration, immune cell infiltration, and extent of tumorigenesis.

#### 2.4.1 Immunohistochemical staining

To assess β‐catenin and Ki67 expression, deparaffinized colonic sections were immersed in pre‐warmed sodium citrate buffer (10 mM tri‐sodium citrate in distilled water containing 0.05% Tween‐20, pH 6.0) for 20 min at 98°C for antigen retrieval. Subsequently, sections were rinsed in PBS three times at 7‐min intervals. The sections were then blocked by normal donkey serum (Sigma‐Aldrich, 10%) containing 0.3% triton‐100 (VWR) for 90 min, followed by three washes in PBS for 8 min. Next, the sections were incubated for 16 h at 4°C in the dark with primary antibodies [β‐catenin (Novus, NBP1‐54467, 1:200) or Ki67 (Novus, NB500‐170, 1:200)] diluted in PBS containing 0.3% triton, 1% BSA, and 1% donkey serum. Sections incubated with diluting buffer without primary antibody were used as negative controls. Following incubation, sections were rinsed in PBS four times with gentle rocking. Then sections were incubated with secondary antibodies [anti‐mouse (Alexa Fluor™ 555, Invitrogen, A‐ A‐48270, 1:400) and anti‐rabbit (Alexa Fluor™ 488, Invitrogen, A‐21206, 1:600) for β‐catenin and Ki67, respectively] for 1 h at room temperature in the dark, followed by four times washing with PBS. Subsequently, the sections were washed and mounted with an aqueous mounting medium containing DAPI and an anti‐fading agent (Sigma Fluoroshield™, F6057). All histology images were generated using Leica THUNDER Imager (Leica DMi8 microscope) and the LAS X software (Leica Microsystems Inc.).

#### 2.4.2 Image quantifications

The colon tumor area was quantified by ImageJ software. The whole colon area was automatically selected by the software via color‐dependent selection. The tumors were manually selected by the predominant circular shape and brightness on the picture. The area was calibrated in centimeters by a ruler present aside in the image of colon tissue.

### 2.5 Quantification of bacteria using quantitative PCR

Quantitative PCR (qPCR) was used to measure the abundance of total bacteria, Firmicutes, Bacteroidota, and *Bifidobacteria*. The QIAamp DNA Stool Mini Kit (Qiagen Inc) was used to extract bacterial DNA from equal amounts of cecal contents. As described in our previous studies,^18^, ^19^, ^24^ cecal bacterial DNA, SYBR green master mix, and bacteria‐specific primers (Table 2) were mixed, and qPCR was performed using the Step One Plus Real‐Time PCR System (Applied Biosystems). 16S rRNA was used as a reference gene to estimate the relative abundance of microbial communities. The data are reported as 2^−∆CCT^.

**TABLE 2:** List of primers used in this study.

Name	Oligonucleotide sequence (5′–3′) forward	Oligonucleotide sequence (5′–3′) reverse	Reference
Universal 16S rRNA	AGAGTTTGATCCTGGCTCAG	CTGCTGCCTCCCGTAGGAGT	Chassaing, 2015^66^
Bacteroidota	CRAACAGGATTAGATACCCT	GGTAAGGTTCCTCGCGTAT	Raetz, 2013^67^
Firmicutes	TGAAACTYAAAGGAATTGACG	ACCATGCACCACCTGTC	Raetz, 2013^67^
*Bifidobacterium genus*	CTCCTGGAAACGGGTGG	GGTGTTCTTCCCGATATCTACA	Murri, 2013^68^

### 2.6 Assessment of microbial metabolites by ^1^H NMR‐based metabolomics

Sample preparation for NMR spectroscopy was performed as previously described in our previous studies.^18^, ^25^ About 50 mg of cecal content was mixed with 600 μL of ice‐cold phosphate buffer (0.1 M K2HPO4/NaH2PO4 = 4/1, pH = 7.4) containing 50% heavy water (D_2_O) and 0.005% (wt/vol) of sodium 3‐ (trimethylsilyl) propionate‐2,2,3,3‐d4 (TSP). Samples were vortexed, homogenized, subjected to a three freeze–thaw cycle in liquid nitrogen, and centrifuged at 14000*g* for 10 min at 4°C. The supernatant was collected in a separate tube, and the remaining pellet was extracted again repeating the above steps. The two supernatants were then combined and vortexed. After centrifuging at 17000*g* for 10 min at 4°C, 550 μL of combined supernatant was transferred to 5 mm NMR tubes for NMR analysis. The 1D ^1^H spectra of cecal extracts were recorded at 298 K by using Bruker Avance NEO 600 MHz NMR spectrometer (operating at 600.15 MHz for proton, Bruker Biospin, Germany) equipped with a 5 mm TCI cryoprobe with enhanced sensitivity for ^1^H and a SampleJet autosampler for high throughput metabolomics. Spectral acquisition parameters were: 48 scans were collected into 64 k data points using 90° pulses, 4 dummy scans, 20 ppm spectral width and 5 s relaxation time. All spectra were analyzed via Chenomx NMR Suite (version 8.4). The analysis consists of processing the raw spectra and profiling the metabolites. After automatic processing, the phase, baseline, and internal standard were checked and modified manually for each spectrum for quality assurance. Metabolite concentrations were exported to an Excel spreadsheet for further statistical analysis.

### 2.7 Human colorectal cancer cell proliferation assay

The human colorectal cancer cell (HCT116) proliferation assay was performed as follows. HCT116 cells were cultured in Dulbecco's modified Eagle's medium (DMEM; Corning) supplemented with 10% fetal bovine serum (FBS; BioFluid), 1% penicillin–streptomycin (Sigma Aldrich), and 0.1% plasmocin® (Invivogen) at 37°C in 5% CO_2_. To assess the effect of succinate on cellular proliferation, HCT116 cells were seeded at the density of 3000 cells per well in 96‐well plates. After 24 h, the cells received fresh media supplemented with different concentrations (vehicle, 0.1 mM or 1 mM) of succinate (Sigma Aldrich) in the presence or absence of an inflammatory cocktail (ICT) containing tumor necrosis factor α [TNFα (25 ng/mL); PeproTech], interferon γ [IFNγ (20 ng/mL); PeproTech], and lipopolysaccharide [LPS (1 μg/mL); Sigma Aldrich]. After 72 h, cell viability was assessed using a modified *Sulforhodamine B* (SRB) assay.^26^ Briefly, cells were fixed with 50% wt/vol trichloroacetic acid (Fisher Scientific) for 1 h at 4°C. The plates were washed with deionized water and dried for ~16 h at room temperature (~24°C). Fifty microliters (50 μL) of 0.04%; SRB dye (Alfa Aesar) was then added to the wells and incubated in the dark for 1 h followed by washing with 1% acetic acid (ChemPure) and incubating in the dark for ~16 h at room temperature (~24°C). Subsequently, 100 μL of 10 mM Tris base buffer (pH 10.5; Fisher Scientific) was added to each well 10 min before the reading at 510 nm using a plate reader.

### 2.8 Statistical Analysis

All data are represented as Mean ± SEM. The normality and equal variance were tested by the Shapiro–Wilk and Bartlett test in RStudio. Statistical significance between the two groups was calculated using an unpaired, two‐tailed *t* test (parametric) or unpaired non‐parametric Mann–Whitney test using GraphPad Prism 9. Data from more than two groups were compared using a one‐way ANOVA followed by Tukey's multiple comparison tests. The significant difference was represented *p* < .05 as *, *p* < .01 as **, *p* < .001 as ***, and *p* < .0001 as ****. Sample sizes of more than three were considered for statistical analysis.

## 3 RESULTS

### 3.1 Inulin exacerbated DSS‐induced colitis

**FIGURE 1:**
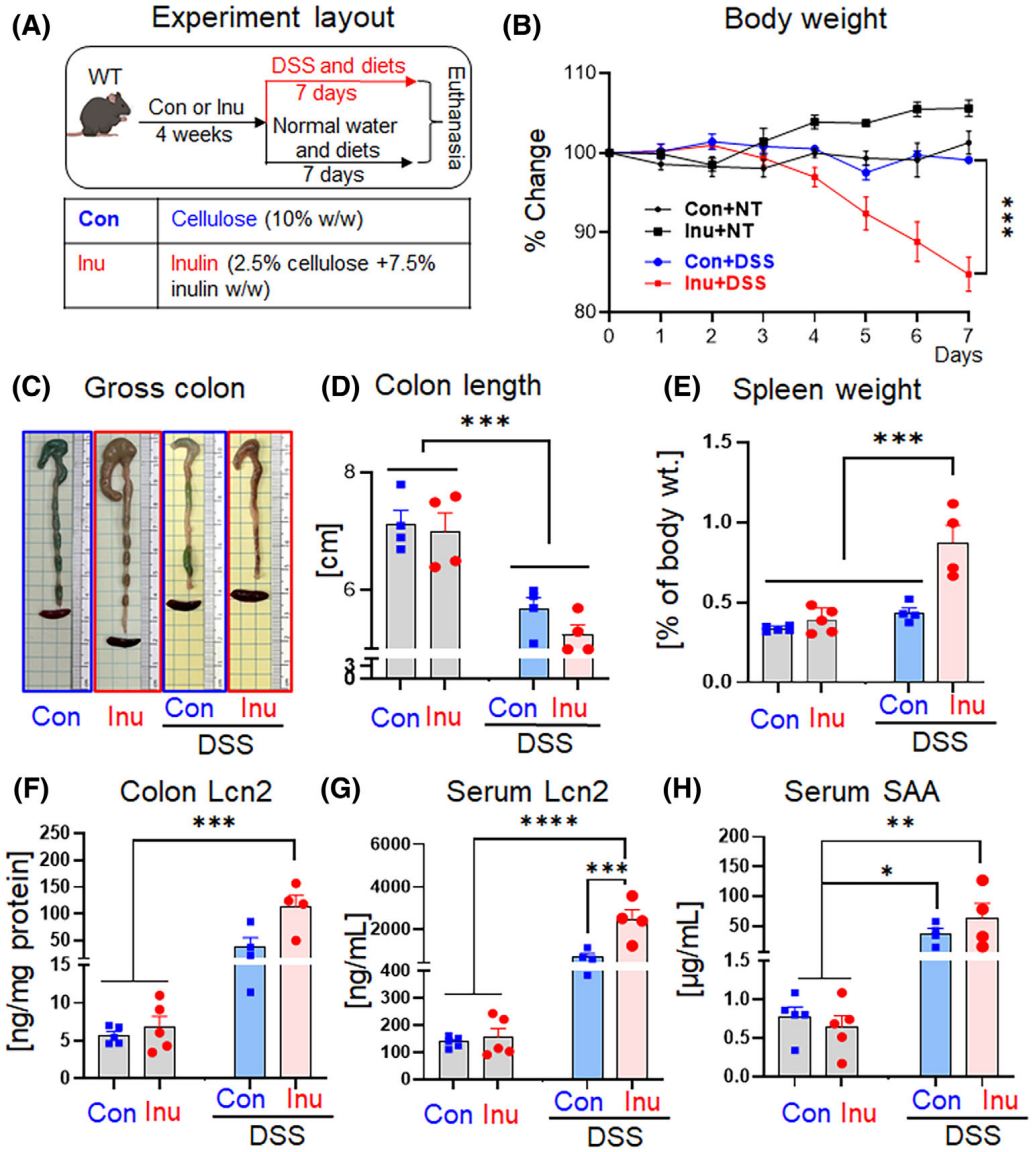
Inulin worsened DSS‐induced colitis. WT mice were fed control (Con) or inulin (Inu) diets for 4 weeks, followed by 1.4% DSS intervention for 7 days. (A) Experiment layout, (B) body weight change during DSS treatment and NT group at the same timeline as DSS group, (C) gross colon and spleen pictures, (D) colon length, (E) spleen weight as percentage of body weight, (F) colon Lcn2, (G) serum Lcn2, (H) serum SAA. Values are shown as mean ± SEM. (B) Unpaired *t* test. (D–H) One‐way ANOVA. *p < .05, **p < .01, and ***p < .001.

Refined inulin, a prebiotic FDF commonly present in processed foods, has been shown to have both beneficial and detrimental effects on intestinal inflammation.^19^, ^20^, ^27^, ^28^, ^29^ Herein, we tested the effect of inulin consumption on colonic inflammation using the DSS model. WT mice were fed with either a cellulose containing diet (control; Con) or an inulin‐containing diet (Inu) for 4 weeks, followed by splitting into two groups receiving drinking water without (no treatment, NT) or with DSS (1.4% wt/vol) (Figure 1A, *n* = 4–6 mice in each group). Body weight was not significantly different in the Con‐NT and Inu‐NT groups. However, the Inu‐DSS group displayed significantly more reduction in body weight than the Con‐DSS group (Figure 1B). Notably, the decrease of body weight in the Inu‐DSS group was apparent as early as 4‐day‐post DSS administration (Figure 1B), indicating the rapid onset of colitis in the Inu‐DSS group than in the Con‐DSS group. Correspondingly, Inu‐DSS mice displayed shortened colon and enlarged spleen after 7 days of DSS intervention (Figure 1C–E). The earlier loss in body weight and shortened colons indicated that the Inu‐DSS mice were experiencing more severe colon inflammation than the con‐DSS group. Relative to NT groups, a trend of an enlarged spleen was observed in the Con‐DSS group, but Inu‐DSS mice displayed marked splenomegaly compared to the other three groups (Figure 1E). Lcn2, a member of the lipocalin family, is one of the widely used disease activity markers for colitis.^30^, ^31^, ^32^, ^33^ Similarly, SAA is another robust marker of both systemic and intestinal inflammation.^34^, ^35^ The levels of Lcn2 and SAA were not significantly different between the Con and Inu groups at the basal level (NT groups). Intriguingly, relative to the basal group, the colonic and serum Lcn2 was significantly elevated only in the Inu‐DSS group but not in the Con‐DSS group, further supporting our observation that inulin exacerbated DSS‐induced colitis (Figure 1F,G). SAA was significantly elevated in both Con‐DSS and Inu‐DSS groups (Figure 1H). Collectively, these data demonstrated that inulin aggravated the DSS‐induced colonic inflammation but did not affect the healthy gut adversely.

### 3.2 Inulin worsened colitis‐associated colorectal tumorigenesis

**FIGURE 2:**
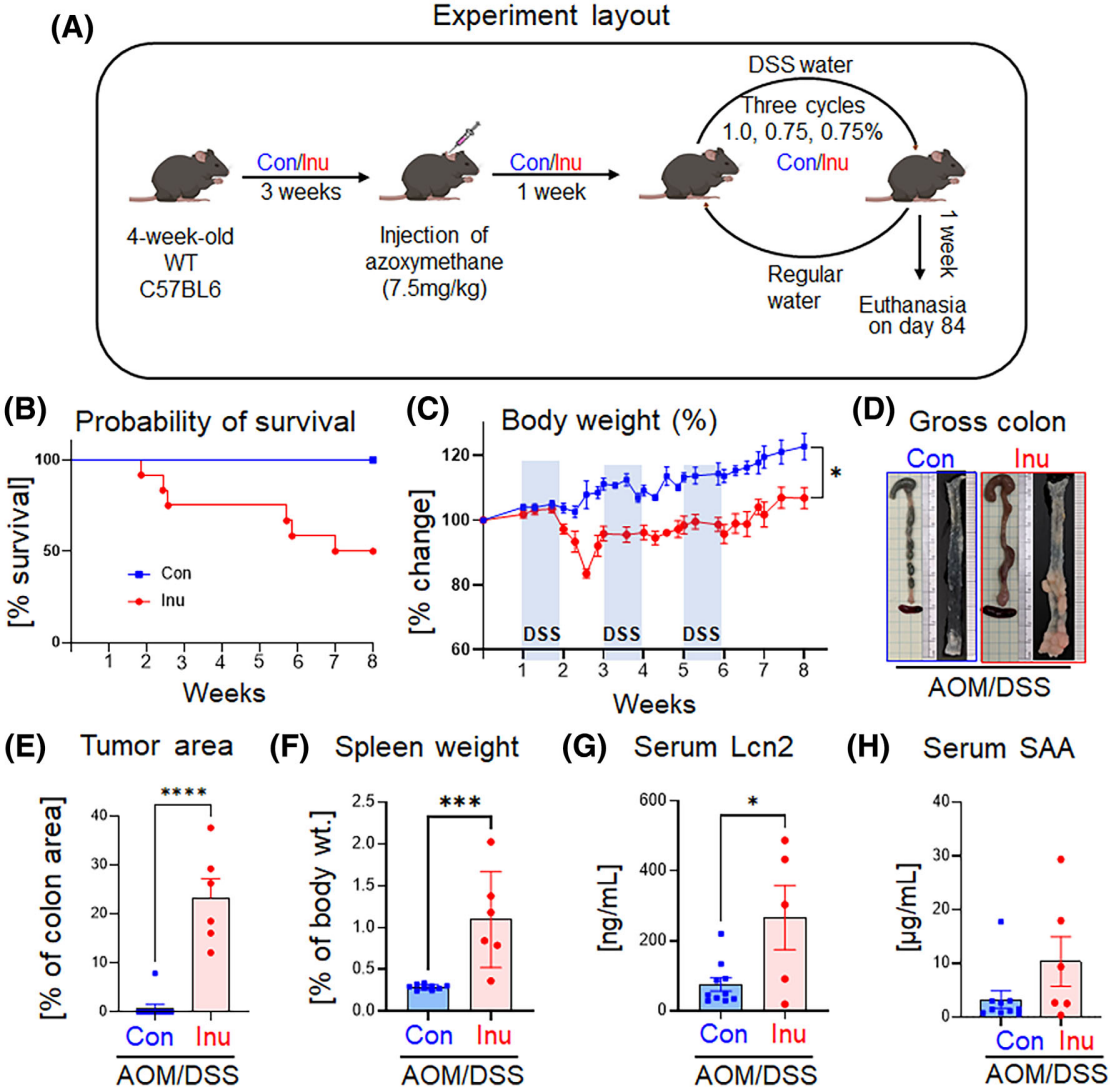
Inulin consumption exacerbated colitis‐associated CRC progression. Mice (*n* = 8–12) were maintained on a control (Con) or inulin (Inu) diet for 3 weeks before azoxymethane (AOM) administration. One‐week post‐AOM injection, DSS in water was provided to all the mice for 7 days, followed by 7 days of regular water. The cycle of DSS/normal water was continued for 6 weeks, 3 cycles in total. Mice were sacrificed 1 week after the last (third) cycle of DSS. (A) Experiment layout, (B) probability of survival of AOM‐treatment mice during DSS/water cycles, (C) body weight change in percentage of the initial weight on AOM injection day, (D) gross colons and longitudinally opened colons illustrating the appearance of tumors, (E) tumor area in percent of the total colon area, (F) spleen weight as the percentage of whole‐body weight on the day of euthanasia. Serum levels of G. Lcn2 and H. SAA. Values are presented as mean ± SEM. (C, E–H) Unpaired *t* test. *p < .05, **p < .01, and ****p < .0001.

Prolonged intestinal inflammation is a major risk factor for CRC development. To expand our understanding of the impact of inulin on intestinal health, specifically inulin‐induced exacerbation of intestinal inflammation on the risk of developing CRC, we used the AOM/DSS model. The combination of DSS with AOM is a well‐established model for colitis‐associated cancer due to its reproducibility, potency, and induction of tumors closely resembling human colorectal cancers.^36^ As outlined in Figure 2A, one‐week post‐AOM injection, the mice received 1% (1 cycle) or 0.75% (2 cycles) DSS in drinking water every other week and consumed regular water in the weeks between each DSS week. Remarkably, a substantial mortality (~50%) during the DSS intervention period was observed in the Inu‐fed group, reinforcing our observation that inulin worsens DSS‐induced colon epithelial injury (Figure 2B). This early death was caused by severe intestinal inflammation and blood loss, as these mice lost body weight very rapidly and had undergone severe diarrhea. As compared to the control mice, the surviving inulin‐fed mice lost more body weight during both the DSS intervention and the normal water‐feeding cycle (Figure 2C). Remarkably, the Inu‐fed group developed extensive colonic tumors (Figure 2D,E). On average, 23.3% of the colon area in these mice was covered by tumors, while only one control mouse developed a colon tumor. The tumor size (7.8% of colon area) was substantially smaller in control than in any mouse in the inulin‐fed group (Figure 2E). Moreover, the spleen weight of the inulin‐fed mice was almost threefold higher than the controls (Figure 2F). In agreement, the inulin‐fed group had a significantly higher level of Lcn2 (Figure 2G) than the controls. However, no difference in serum SAA was evident (Figure 2H). Collectively, we observed that the Inu‐fed group exhibited extensive morbidity and colon tumorigenesis, indicating that inulin consumption predisposed mice to develop CRC.

**FIGURE 3:**
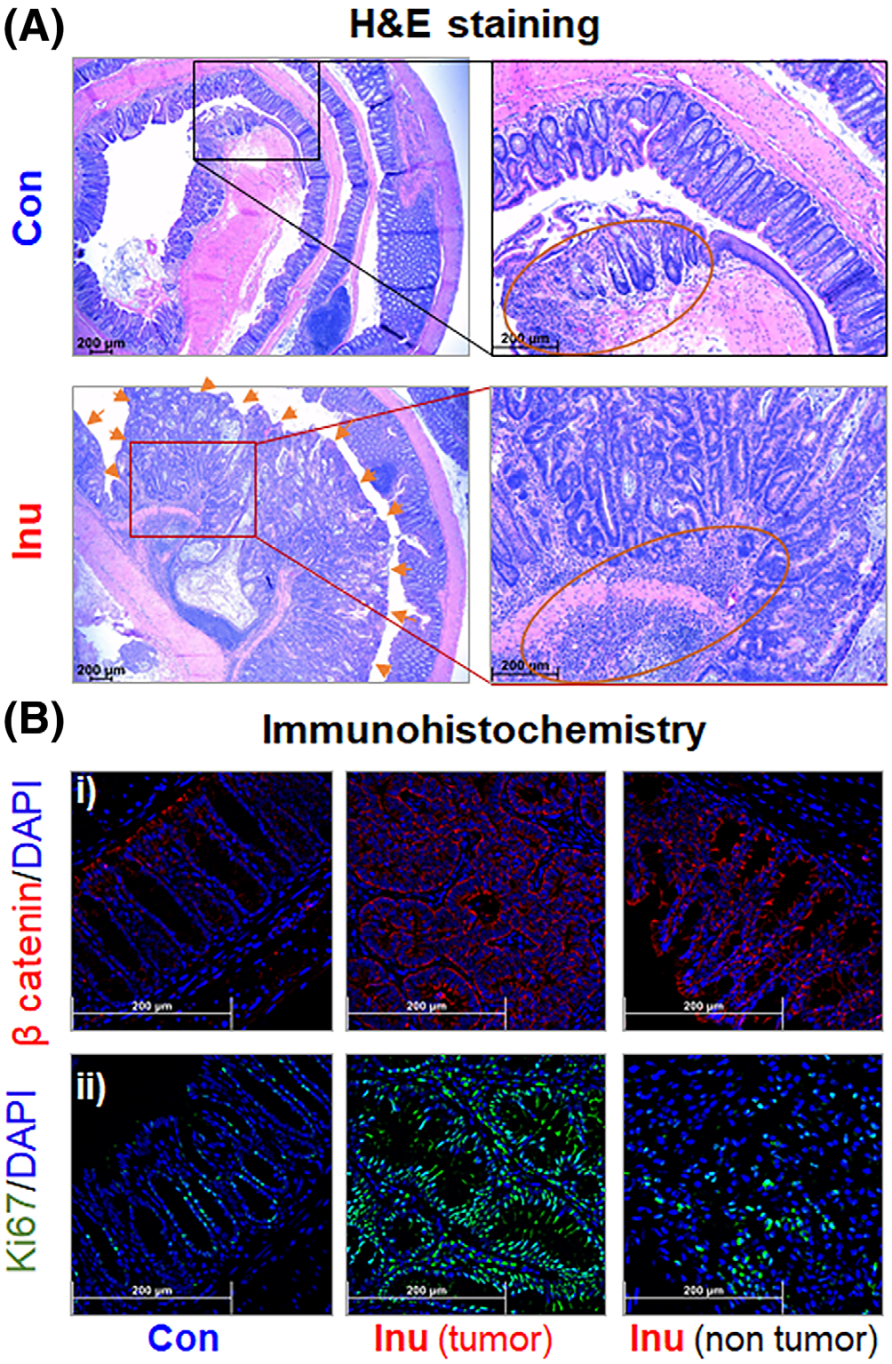
Apparent colon tumors with higher intracellular β‐catenin accumulation and cell proliferation were observed in the inulin‐fed group. Representative images of colon sections were obtained from the AOM/DSS treatment group. (A) H&E staining of colon sections at ×16 (left) and ×50 (right) magnification. Arrows denote colon tumor. Red circles show colon architecture disruption and immune cell infiltration. (B) immunohistochemical staining for (i) ß‐catenin (red) and (ii) Ki67 (green) at ×400 magnification.

Histological analysis of the H&E‐stained colon revealed apparent tumors in the inulin‐fed group (Figure 3A). To characterize the colon tumors, we next performed immunostaining for ß‐catenin and Ki67. The Wnt/ß‐catenin signaling pathway plays a critical role in cell proliferation and homeostasis of intestinal epithelial cells; thus, it is widely used as a marker of colon tumorigenesis.^37^, ^38^ The internalization of ß‐catenin, from the cell membrane to cytosol and nucleus, is linearly correlated with the progression stages of colon carcinoma.^39^ Herein, immunostaining showed that ß‐catenin was largely located on the epithelial cell membrane in the control group (Figure 3Bi). In contrast, cytosolic and nuclear ß‐catenin were detected in the Inu group, suggesting reduced degradation of ß‐catenin and upregulation of the Wnt signaling pathway (Figure 3Bi). Besides ß‐catenin, Ki67 is another prognostic marker in colon cancer that is highly expressed in proliferating cells in the cell cycle of G1‐M phases.^40^ Immunohistochemical analysis in the colon tissues from both groups showed that Ki‐67 was predominantly expressed in the tumor regions of inulin‐fed groups (Figure 3Bii). The overall evidence demonstrated that refined inulin, compared with the control group, aggravated mouse colon tumorigenesis.

### 3.3 Inulin elevated luminal succinate and supplementation of succinate promoted human colorectal carcinoma cell proliferation

**FIGURE 4:**
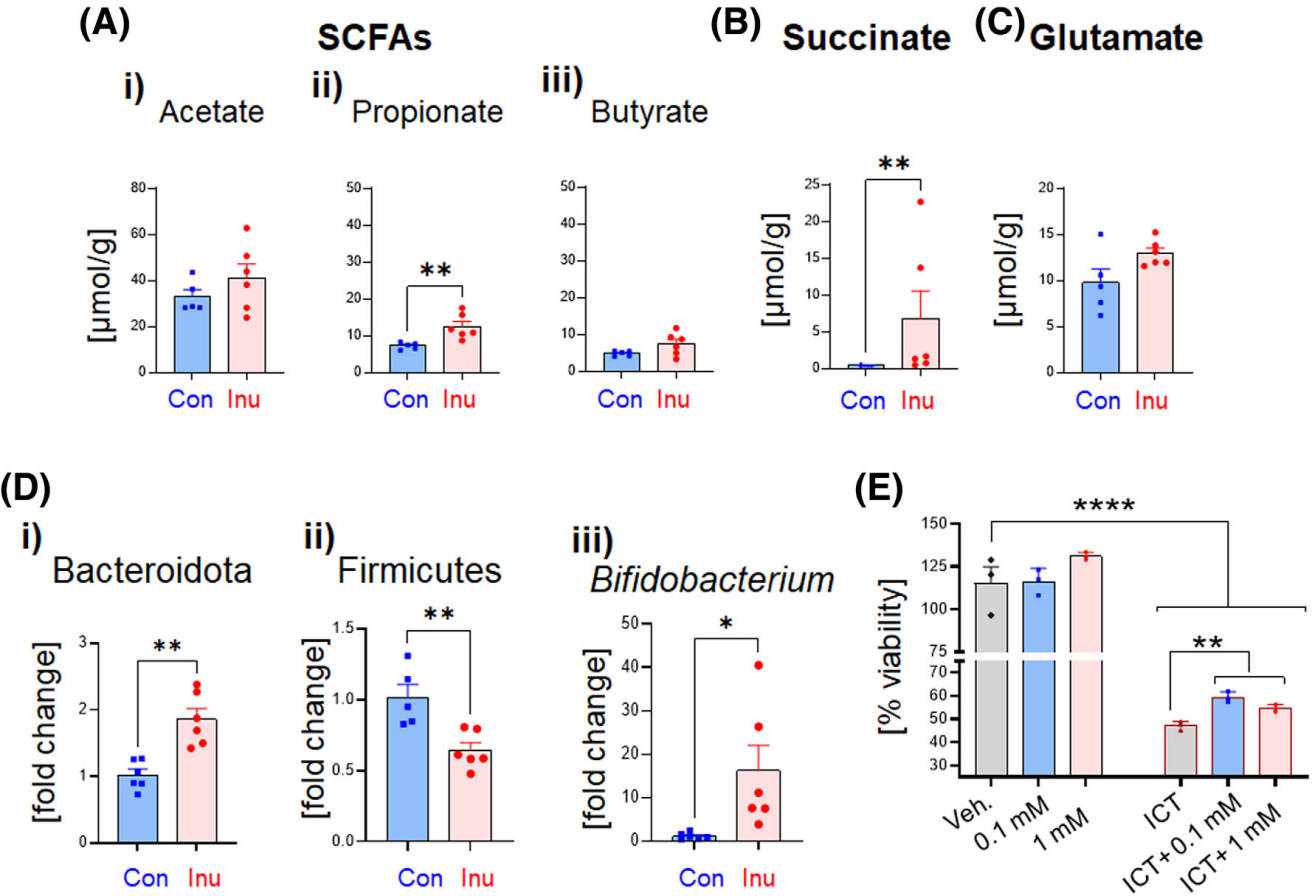
Inulin‐induced changes in gut microbiota increased luminal succinate levels, and the presence of succinate enhanced the proliferation of human colorectal carcinoma cells. WT mice (*n* = 6) were maintained on control or inulin‐containing diets for 5 weeks, and cecal contents were collected upon euthanasia for metabolomic investigation using ^1^H‐NMR. Bar graphs represent cecal levels of (A) short‐chain fatty acids (SCFAs): (i) acetate, (ii) propionate, and (iii) butyrate, (B) succinate, and (C) glutamate. Cecal content derived from control or inulin‐fed mice were used to characterize the gut microbiota via qPCR. (D) Relative cecal abundance of phyla (i) Bacteroidota and (ii) Firmicutes, and genus (iii) *Bifidobacterium*. Human colorectal carcinoma cell line (HCT116) was treated with succinate (0.1 mM or 1 mM) in the presence or absence of an inflammatory cocktail [ICT, a mixture of TNFα (25 ng/mL), IFNγ (20 ng/mL) and LPS (1 μg/mL)], in triplicates, and assessed for cell viability/proliferation until 72 h. **E.** Percent (%) cell viability. Values are presented as mean ± SEM. (A and C‐D) Unpaired *t* test, (B) Unpaired non‐parametric Mann–Whitney test. (E) One‐way ANOVA. *p < .05, **p < .01, and ****p < .0001.

The extracellular accumulation of oncogenic signaling molecules, such as succinate, fuel the initiation and progression of carcinogenesis.^41^, ^42^ Both host cells and gut bacteria produce succinate; however, in the intestinal lumen succinate is chiefly derived from bacterial metabolism.^43^ Short‐chain fatty acids (SCFAs) that include acetate, propionate, and butyrate are another abundant product of microbial fermentation in the gut lumen. The ^1^H‐NMR‐based metabolomic analysis of cecal contents revealed a marked elevation of cecal propionate with an increasing trend in acetate and butyrate in the inulin group than the control (Figure 4Ai–iii). Intriguingly, cecal level of succinate was significantly augmented in the Inu‐fed group (Figure 4B,C). Bacterial species that are known to regulate luminal succinate level belong to phyla Bacteroidota and Firmicutes. Notably, we observed an increase in the cecal abundance of  Bacteroidota in inulin‐fed mice (Figure 4Di). Intriguingly, the abundance of Firmicutes reduced in response to inulin intervention (Figure 4Dii). Collectively, this data suggests that the inulin‐induced shift in gut microbiota supported the elevation of succinate in the gut lumen. Of note, we did observe a disproportionate enrichment of genus *Bifidobacterium*, which belong to the phylum Actinobacteria in inulin‐fed mice (Figure 4Diii). More importantly, we observed increased proliferation of human colorectal carcinoma cells (HCT116) in response to succinate treatment (Figure 4E) in the presence of an inflammatory cocktail [ICT, a mixture of TNFα (25 ng/mL), IFNγ (20 ng/mL) and LPS (1 μg/mL)], but not at the basal level, indicating that succinate fuels cellular proliferation in the inflammatory environment. Altogether, this data suggests that the accumulation of succinate in the intestinal lumen partly contributes to cancer development in the inflamed gut.

## 4 DISCUSSION

This study investigated the effect of refined FDF inulin on mucosal inflammation and colitis‐associated CRC development. We found that inulin consumption predisposed mice to colonic inflammation. Unexpectedly, ~50% of the inulin‐fed mice died in the colitis‐associated CRC model (AOM/DSS), possibly due to impaired mucosal healing and higher inflammation. Notably, the inulin‐fed group exhibited extensive colon tumor load, whereas the control group had minimal apparent colon tumors. Importantly, inulin consumption did not alter the markers of colonic inflammation in the healthy mice but elevated luminal succinate, an inflammatory and oncogenic metabolite,^44^, ^45^ partly by augmenting the abundance of succinate‐producing bacteria in the distal gut. Increased proliferation of human colorectal carcinoma cells upon co‐treatment with inflammatory protein and succinate indicates that inulin‐induced elevation of luminal succinate partly contributed to colon tumorigenesis.

The incidence of IBD and CRC are markedly higher in high‐income countries and are increasing in developing countries.^46^, ^47^ Most CRCs are observed in adults after 50 years old in the United States, but early‐onset CRC in youth is emerging.^48^ Based on the projection of aging and population growth, the study by Xi et al., predicted that the global number of CRC cases would increase approximately from 1.9 million to 3.2 million by 2040.^46^ Such escalating trends of CRC warrant the identification of the factors contributing to CRC development. Our study suggests that refined inulin may increase the risk of colon cancer in susceptible individuals.

Unprocessed FDFs found naturally in fruits and vegetables benefit intestinal health predominantly by maintaining microbial diversity in healthy individuals.^49^, ^50^ Based on the long‐standing notion that naturally occurring DFs benefit gut health, food manufacturers advocate consuming processed FDFs such as inulin. However, whether such highly processed FDFs hold physiological effects similar to their naturally occurring counterparts remains unclear. Studies have yielded conflicting evidence on whether refined FDFs attenuate or deteriorate IBD development.^19^, ^20^, ^51^, ^52^ For example, fermentable oligosaccharides, disaccharides, monosaccharides, and polyols (FODMAPs) were shown to exacerbate clinical complications in a subgroup of IBD patients.^53^, ^54^ Moreover, the intake of DF was beneficial to reduced flares in patients with Crohn's disease but not in patients with UC.^55^ The effects of FDFs in the context of IBD‐associated colorectal cancer are also poorly understood. Whole grain fibers are found protective against CRC in the NIH‐AARP Diet and Health Study cohort.^56^ However, the effect of refined DFs on CRC initiation and progression is still unclear. The experimental findings suggest a protective^57^, ^58^, ^59^ or minimal to no effect^60^, ^61^ of inulin or inulin‐derived fructo‐oligosaccharide supplementation on the risk factors of CRC. This ambiguity is possibly due to the substantial reduction in richness and fiber fermentation capacity of microbiota residing in the inflamed and tumorigenic environment. The beneficial potential of dietary fibers is primarily deduced from studies involving healthy individuals; we propose that the response of FDFs on host intestinal health is impacted by pre‐existing dysbiosis, as in the inflamed gut. In support, we found no protection with the isolated FDF inulin in our previous^19^ and in the present study; in contrast, it worsened colonic inflammation and augmented colon tumorigenesis.

An intricate relationship between FDFs, gut bacteria, and intestinal immune cells helps to fine‐tune intestinal immune response and prevent chronic intestinal inflammation. We theorize that regular consumption of an ultra‐processed single type of DF affects such complex networks of interactions detrimentally by promoting the growth of a selective group of bacteria. For example, supplementation of inulin preferentially increases the proportion of *Bifidobacterium*.^62^ A recent study by Wei et al.^63^ found that *Bifidobacterium* was significantly increased in the colon biopsy specimens of active UC patients compared to those in healthy controls. In this study, the authors suggested a cautious use of probiotics containing *Bifidobacterium* in IBD patients during the active phase of the disease as such a disproportionate increase of mucosal *Bifidobacterium* could contribute to the IBD flare‐up. In agreement, we found more than ~15 folds expansion of *Bifidobacterium* in mice maintained only on the inulin containing diet. Inulin is known to increase SCFAs that are considered beneficial for intestinal health. In the present study, inulin‐fed mice displayed elevated levels of cecal propionate with an increasing trend in acetate and butyrate; however, such enrichment of these SCFAs failed to protect against colonic inflammation and tumorigenesis in inulin‐fed mice. Notably, we found elevated luminal level of inflammatory and oncogenic metabolite succinate^44^, ^45^ in response to inulin intervention. Such an increase in cecal succinate correlated with the expansion of the phylum Bacteroidota, which contains major producers of succinate in the mammalian gut.^64^

Collectively, inulin‐induced changes in gut microbiota composition and metabolic activity were associated with exacerbated DSS‐induced colitis, delayed recovery, and mortality (~50% of inulin‐fed mice succumbed to death in AOM/DSS treatment group). Notably, no death or a worsened colitis phenotype (abrupt loss in body weight and severe bloody diarrhea) was observed in the control (non‐fermentable fiber) group. More importantly, all the mice that survived from the inulin group, but not in the control group, exhibited extensive colorectal tumorigenesis. We surmise that in contrast to inulin present in whole fruits and foods, processed forms of inulin fuel selective bacterial colonization (e.g., expansion of *Bifidobacterium*) that prolongs and worsens colonic inflammation and increases colon tumorigenesis. However, these speculations need to be tested through detailed characterization of gut microbiota and their metabolites in samples derived from both healthy and inflamed gut. The limitations of our study include the lack of detailed compositional and functional characterization of gut microbiota through metatranscriptomic analysis. Incorporating functional pathway analysis such as metatranscriptomics will help to identify the microbial metabolism pathways that contributed to augmenting luminal succinate in the inulin‐containing diet‐fed group. We have examined the effect of inulin on two major bacterial phyla, Firmicutes and Bacteroidota and genus *Bifidobacterium*, and a specific shift in microbial metabolism. However, whether such inulin‐induced alterations in gut microbiota activity contributed to extensive colon tumorigenesis has also not been elucidated. We propose testing it with well‐designed studies, including microbiota transplantation in germ‐free mice. An additional limitation of our study is using an inflammation‐dependent colon cancer model. Such inflammation dependency of the AOM/DSS model limits us from identifying whether inulin‐gut microbiota axis‐derived products have an independent effect on colon cancer development as inulin exacerbated colonic inflammation.

IBD patients present a 3–5‐fold increase in the risk of developing CRC.^65^ Atypical changes in gut microbiota composition and activity increase the propensity to develop CRC by prolonging intestinal inflammation and creating a tumorigenic environment. Thus, identifying dietary components, including FDFs, that impact gut microbiota unfavorably and fuel intestinal inflammation will allow IBD patients to make informed dietary choices and help to reduce the risk of CRC development. In line, present study advances our understanding by demonstrating that isolated FDF, inulin, potentiates colonic inflammation and colitis‐associated colon tumorigenesis in mice. Therefore, avoiding the consumption of isolated refined inulin may help reduce the risk of colon cancer in patients with IBD in the long run.

## AUTHOR CONTRIBUTIONS

Vishal Singh conceived the project. Sangshan Tian and Vishal Singh designed the experiments, analyzed the data, and wrote the manuscript. Sangshan Tian conducted all the mouse experiments. Devendra Paudel assisted with mouse experiments and performed histological analysis and imaging. Fuhua Hao and Andrew D. Patterson analyzed the metabolomics data and provided expertise in data interpretation. Rabin Neupane and Amit K. Tiwari were instrumental in assessing colonocyte proliferation and provided expertise in data interpretation. Rita Castro assisted with colon inflammation marker analysis. K. Sandeep Prabhu provided expertise on colitis and colon carcinogenesis marker analysis and interpretation. Amit K. Tiwari, Andrew D. Patterson, Rita Castro, and K. Sandeep Prabhu provided feedback on data analysis and manuscript writing. All authors have read and agreed to the published version of the manuscript.

## CONFLICT OF INTEREST STATEMENT

The authors have stated explicitly that there are no conflicts of interest in connection with this article.

## ETHICS STATEMENT

This study was approved by the Institutional Animal Care andUse Committee (IACUC) of The Pennsylvania State University.

## Data Availability

Data sharing is not applicable to this article as no new data were created or analyzed in this study.
